# New species and new records of *Artabotrys* (Annonaceae) from peninsular Thailand

**DOI:** 10.3897/phytokeys.151.51643

**Published:** 2020-06-17

**Authors:** Junhao Chen, Wichan Eiadthong

**Affiliations:** 1 Singapore Botanic Gardens, National Parks Board, 1 Cluny Road, 259569, Singapore Singapore Botanic Gardens Singapore Singapore; 2 Faculty of Forestry, Kasetsart University, Bangkhen campus, Jattujak, Bangkok, Thailand Kasetsart University Bangkok Thailand

**Keywords:** Annonaceae, *
Artabotrys
*, new records, new species, peninsular Thailand

## Abstract

Two new species of *Artabotrys* (Annonaceae) are described from peninsular Thailand. *Artabotrys longipetalus* J.Chen & Eiadthong, **sp. nov.**, is unique among *Artabotrys* species in Thailand in having linear petals, relatively long flower pedicels and sessile monocarps. *Artabotrys insurae* J.Chen & Eiadthong, **sp. nov.**, resembles *Artabotrys uniflorus* (Griff.) Craib, but can be distinguished by its oblique leaf base, flat petal blades, apiculate anther connective apex and the presence of a monocarp stipe. In addition, two new records for the Flora of Thailand are reported, viz. *Artabotrys crassifolius* Hook.f. & Thomson and *Artabotrys pleurocarpus* Maingay ex Hook.f. & Thomson; both species are so far only known from peninsular Thailand. A key to the 20 species of *Artabotrys* in Thailand is provided.

## Introduction

*Artabotrys* R.Br. (Annonaceae) is a palaeotropical genus of woody climbers that inhabits tropical rain forests and seasonally dry forests. The genus comprises over 100 species, with the majority occurring in Asia, ca. 30 species in Africa, and one species in Northern Australia (Chen et al. 2018). The presence of specialised inflorescence hooks that assist climbing distinguishes *Artabotrys* from other Annonaceae climbers. A recent molecular phylogenetic study (Chen et al. 2019) revealed that the genus consists of an early-divergent grade (EDG) of two African species, and a main *Artabotrys* clade (MAC) comprising an Asian clade sister to an African clade. *Artabotrys* possess trimerous flowers, with a whorl of sepals and two whorls of petals. The outer and inner petals are generally similar in size whereas the sepals are much smaller than the petals. MAC species are characterised by petals with a distinct upper blade and a basal concave claw, and an elaborate rim between the inner petal blade and claw which enables the inner petals to cohere tightly over the reproductive organs (Chen et al. 2020). Conversely, EDG species lack a projecting rim on the inner petals, with one species (*A. brachypetalus* Benth.) entirely lacking the distinction between petal blade and claw (Chen et al. 2020). The flowers of *Artabotrys* are hermaphroditic, with many stamens and few to many unfused carpels. Each carpel has two ovules on a basal placenta. After fertilisation, these carpels develop into free monocarps that are sessile or borne on short stipes.

**Figure 1.**
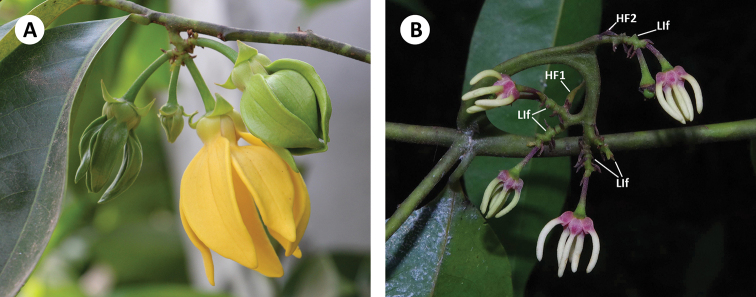
**A***Artabotrys hexapetalus*, with condensed lateral inflorescence branches borne on a peduncle that barely resembles a hook **B***Artabotrys suaveolens*, with elongate lateral inflorescence branches (LIf) borne on a conspicuously hooked peduncle. HF1: Hook leaf 1; HF2: Hook leaf 2 (*H. Sauquet HS164*). Photos: **A** J. Chen **B** T.L.P. Couvreur.

**Figure 2.**
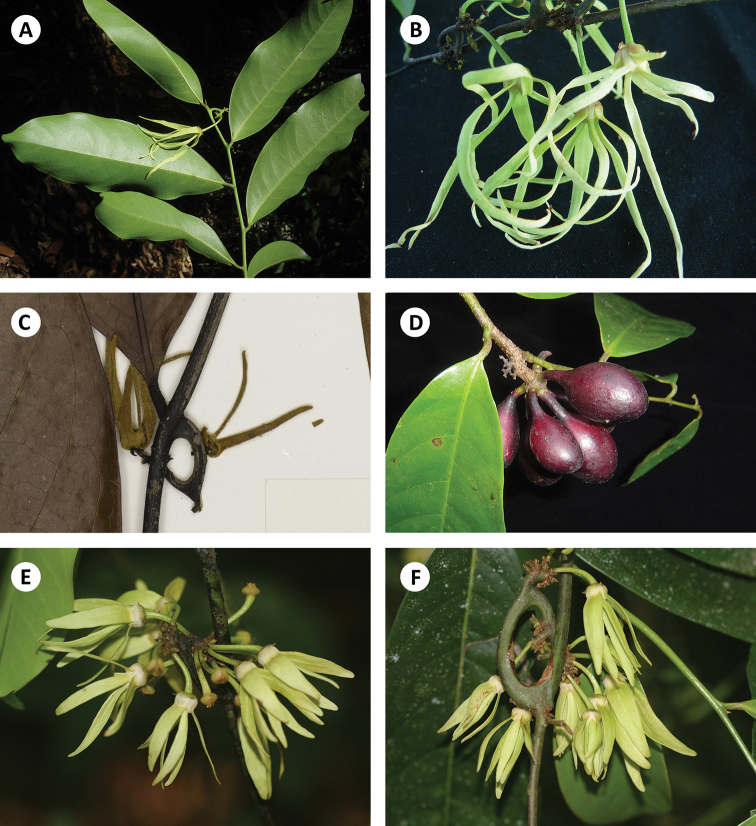
**A, B***Artabotrys longipetalus* sp. nov. **A** habit and inflorescence (*T. Insura 57*) **B** flowers, showing linear petals with acute apex (*T. Insura 57*) **C, D***Artabotrys tipuliferus***C** flowers, showing short flower pedicel and linear petals (*S. Phusomsaeng 272*) **D** fruit, showing short fruit pedicel and stipitate monocarps (*T. Insura 56*) **E, F***Artabotrys multiflorus***E** flowers, showing lanceolate petals with acute to obtuse apex **F** hooked inflorescence with many flowers. Photos: **A, B, D** T. Insura **C** Royal Botanic Garden Edinburgh **E, F** P. Chalermglin.

Herbarium specimens are easily assigned to *Artabotrys* if the diagnostic inflorescence hooks are present. The inflorescence is sometimes only slightly recurved, however; in rare cases, it does not manifest as a hook (Fig. 1A). The inflorescence hook is peculiar with regard to its morphology and ontogeny, which are discussed in great detail in Posluszny and Fisher (2000). The hook formation involves successive development of two hook leaves (hook leaf 1, HL1 followed by hook leaf 2, HL2), flattening and curving of the inflorescence away from the main shoot, and curving of the inflorescence back towards the main shoot. Uneven tissue expansion displaces HL_1_ to the distal position and HL_2_ to the proximal position (figs 23, 24 in Posluszny and Fisher 2000). The floral bud in the axil of HL_2_ is in fact the original apical meristem. The definition of the peduncle, which is usually the distance between the first-formed lower bract and the twig, is contentious in *Artabotrys* because the first-formed bract (HL_1_) is not in the lowest (proximal) position. Here, we regard the entire hook (curved axis from HL_1_ to twig) as the peduncle but it should be noted that its morphology is highly variable within a species, becoming woody as it clasps onto a twig or not manifest as a hook at all as mentioned earlier. Further higher-order branching of the hook inflorescence may occur, resulting in the formation of lateral branches, which are defined as the axes between the hook and the base of the last pedicel (Fig. 1B). The inflorescence lateral branches may be condensed (Fig. 1A, 2F) or elongate (Fig. 1B). Although the genus is easily recognised, identification at the species level is not as straightforward. Petal morphology is sometimes useful (especially for fresh material), but a suite of more subtle characters are often needed for the identification of herbarium specimens. The characters of taxonomic utility in *Artabotrys* include indumentum on lower leaf surface (erect vs. appressed), leaf base (cuneate vs. decurrent on petiole vs. rounded vs. oblique), pedicel length, sepal size, petal size and shape, anther connective apex (apiculate vs. truncate), number of carpels per flower, number of monocarps per fruit, monocarp apex, monocarp stipe length and monocarp size.

Although Thailand is considered to be well known botanically, there remains an upward trend in the number of plant species described from Thailand (Middleton et al. 2019). This is also the case for Annonaceae, particularly in peninsular Thailand where new Annonaceae species are continuously added to the baseline of 39 species listed in Craib (1925). Over the past five years, for instance, an *Alphonsea* species (Turner and Utteridge 2017), two *Artabotrys* species (Turner and Utteridge 2015; Chen et al. 2018), a *Meiogyne* species (Johnson et al. 2019), two *Mitrephora* species (Damthongdee et al. 2019; Saunders and Chalermglin 2019) and two *Xylopia* species (Johnson and Murray 2019) were recently reported as new to science and narrowly distributed in peninsular Thailand and/or northern Peninsular Malaysia. During the preparation of a revision of *Artabotrys* for the Flora of Thailand, two new species and two new records from peninsular Thailand are discovered and reported here, bringing the total number of species recognised for Thailand to 20 (including the commonly cultivated *A. hexapetalus* (L.f.) Bhandari). A key to the 20 species of *Artabotrys* in Thailand is provided.

## Material and methods

The material studied include herbarium specimens of *Artabotrys* from Thailand and neighbouring regions housed in various herbaria (A, BK, BKF, L, PSU, QBG and SING) and digital images of specimens (especially types) from JSTOR Global Plants (https://plants.jstor.org/) and other online herbarium databases viz. AAU (https://www.aubot.dk/search_form.php), BM (https://data.nhm.ac.uk/dataset/collection-specimens), E (https://data.rbge.org.uk/search/herbarium), K (https://apps.kew.org/herbcat/navigator.do), L (https://bioportal.naturalis.nl) and P (https://science.mnhn.fr/institution/mnhn/collection/p/item/search). All specimens cited in this paper have been seen. All measurements were taken from herbarium specimens.

The conservation status of the new species was assessed using the criteria stipulated in the IUCN Red List (IUCN 2012). The extent of occurrence (EOO) and area of occupancy (AOO) of each new species were calculated with the default 2 km^2^ grid using GeoCAT (Bachman et al. 2011; http://geocat.kew.org). The abbreviations used in the conservation assessments follow IUCN (2012).

## New species descriptions

### Artabotrys longipetalus

Taxon classificationPlantaeMagnolialesAnnonaceae

J.Chen & Eiadthong sp. nov.

6809B213-D76B-5319-9174-D68D78CDAF97

urn:lsid:ipni.org:names:77209924-1

Fig. 2A, B

#### Diagnosis.

Distinct from *A. tipuliferus* I.M.Turner & Utteridge (Fig. 2C, D), the only other *Artabotrys* species in Thailand with linear petals, by its relatively long flower pedicels (8–16 mm long vs. 2–4 mm long) and sessile (vs. stipitate) monocarps. Similar to *A. multiflorus* C.E.C.Fisch. (Fig. 2E, F) but distinguished by its chartaceous (vs. coriaceous) leaves, acute (vs. obtuse to acute) petal apex and linear (vs. narrowly lanceolate) petals, specifically its longer and narrower outer petals (35–45 mm long, blade 1–2 mm wide vs. 18–30 mm long, blade 3–5 mm wide) and inner petals (32–40 mm long, blade 1–1.5 mm wide vs. 18–27 mm long, blade 2–4 mm wide).

#### Type.

Peninsular Thailand. Surat Thani Province: Ban Na San District, Tai Rom Yen National Park, Dat Fa Waterfall, 730 m elev., 25 February 2006, *S. Gardner ST2374* (holotype: BKF [SN 198209]; isotypes: BKF [SN 198210], QBG [SN 49402]).

#### Description.

Climbers, to ca. 10 m tall. Twigs drying light brown to brownish black, glabrous, epidermis non-flaky. Leaf laminas 8.5–15 cm long, 2.9–7.7 cm wide, elliptic to oblong-elliptic, chartaceous, glabrous above and below; base cuneate or decurrent on petiole; apex acute to acuminate, acumen up to 5 mm long; midrib raised to flush above, prominent below; secondary veins 7–12 pairs per leaf, raised to flush above and below; tertiary venation reticulate, visible on both surfaces; petiole 2–8 mm long, 1–1.5 mm in diameter, glabrous. Inflorescences 1–15-flowered, peduncles recurved (often laterally compressed and hook-like), glabrous, lateral branches condensed, pedicels 8–16 mm long, ca. 1 mm in diameter, subglabrous. Sepals 3, free, valvate, ca. 1.5 mm long, 1.5–2 mm wide, ovate, glabrous inside, sparsely puberulent outside, apex acute, green in vivo. Petals 6, free, valvate, sparsely appressed-pubescent to glabrous on both surfaces except the glabrous base inside, membranous, greenish yellow in vivo, blade often curly, base concave. Outer petals 3, 35–45 mm long, claw 2–2.5 mm wide, blade 1–2 mm wide, linear, apex acute. Inner petals 3, 32–40 mm long, claw 1.5–2 mm wide, blade 1–1.5 mm wide, linear, apex acute. Stamens 25–35, ca. 1 mm long, ca. 1 mm wide, oblong, anther connective apex truncate. Carpels 8–10, ovary ca. 1 mm long, ca. 0.5 mm wide, stigma ca. 0.5 mm long, cylindrical. Fruit of up to 8 monocarps borne on a glabrous pedicel 19–22 mm long, ca. 4 mm in diameter. Monocarps ca. 26 mm long, 18–20 mm wide, broadly ellipsoid, rough, glabrous, apex weakly beaked (ca. 1 mm long) or rounded, sessile, colour in vivo unknown, drying brownish black, pericarp thickness unknown. Seeds not seen.

#### Phenology.

Flowering specimens collected in February and August; fruiting specimens collected in May.

#### Distribution and habitat.

So far only known from peninsular Thailand (Fig. 6). It occurs in lowland rain forests at elevation 100–730 m, in both undisturbed and partially disturbed sites, sometimes along ridges.

**Etymology.** The specific epithet reflects the long petals of this species.

#### Preliminary conservation status.

*Artabotrys longipetalus* is only known from three localities, with estimated EOO and AOO of 1,165 km^2^ and 12 km^2^, respectively. All the localities are well within the boundaries of various National Parks in Thailand. Nevertheless, this species may become threatened with future climate change and/or other unpredictable threats owing to its restricted AOO and few known locations. Therefore, we suggest a status of Vulnerable [VU D2].

#### Additional specimens examined.

Peninsular Thailand. Nakhon Si Thammarat Province: Lan Saka District, Khao Luang National Park, Karom Waterfall, 100 m elev., 11 August 2006, *T. Insura 57* (BKF). Surat Thani Province: Vibhavadi District, Kaeng Krung National Park, ridge ca. 2 km east of Ban Cham village, 200 m elev., 13 May 2006, *S. Gardner & P. Sidisunthorn ST2731* (BKF).

#### Notes.

*Artabotrys longipetalus* is similar to *A. multiflorus* from Myanmar (Dawna Range) and Thailand (Kanchanaburi and Tak) in having long, narrow petals and sessile monocarps with rounded to weakly beaked apex. Also comparable to the new species, *A. arachnoides* J.Sinclair from New Guinea shares a similar floral morphology of long, linear, curly petals and long flower pedicels but differs in its highly coriaceous leaves, larger sepals (4–5 mm long) and larger petals (50–60 mm long, 2–3 mm wide). A number of *Artabotrys* species also possess linear petals but have very short flower pedicels (2–4 mm long): *A. speciosus* Kurz from the Andaman Islands, *A. sumatranus* Miq. from Sumatra, Java and Borneo (Kalimantan), and *A. tipuliferus* from peninsular Thailand and Peninsular Malaysia. Significantly, *A. longipetalus* was previously confused with *A. sumatranus* in Insura (2009). “*Artabotrys sumatranus*” in Insura (2009) consists of mixed elements: the description and line drawing of the vegetative parts and flowers match *A. longipetalus* whereas the description and line drawing of the fruit match *A. tipuliferus*. Apart from the short flower pedicels, *A. tipuliferus* can be further distinguished from the new species by its apiculate anther connective apex and the presence of a monocarp stipe (ca. 1 cm long) whereas *A. sumatranus* is distinct from the new species in having shorter petals (up to 15 mm long) and apiculate anther connective apex.

### Artabotrys insurae

Taxon classificationPlantaeMagnolialesAnnonaceae

J.Chen & Eiadthong sp. nov.

F40C4959-8AAB-50F4-8487-D04024F59F34

urn:lsid:ipni.org:names:77209925-1

Fig. 3 , 4A–C

#### Diagnosis.

Distinct from other *Artabotrys* species in Thailand in having oblique leaf base and erect-pubescent lower leaf surface. Similar to *A. uniflorus* Craib (Fig. 4D–F) but with oblique (vs. rounded or rarely cuneate) leaf base, flat (vs. three-angled) petal blades, apiculate (vs. truncate) anther connective apex and short-stipitate (vs. sessile) monocarps that are weakly beaked (beak ca. 1 mm long vs. 2–5 mm long).

#### Type.

Peninsular Thailand. Surat Thani Province: Vibhavadi District, Khlong Yan Wildlife Sanctuary, trail from headquarters, ca. 200 m elev., 31 August 2002, *D.J. Middleton et al. 1487* (holotype: BKF [SN 142020]; isotype: A).

**Figure 3.**
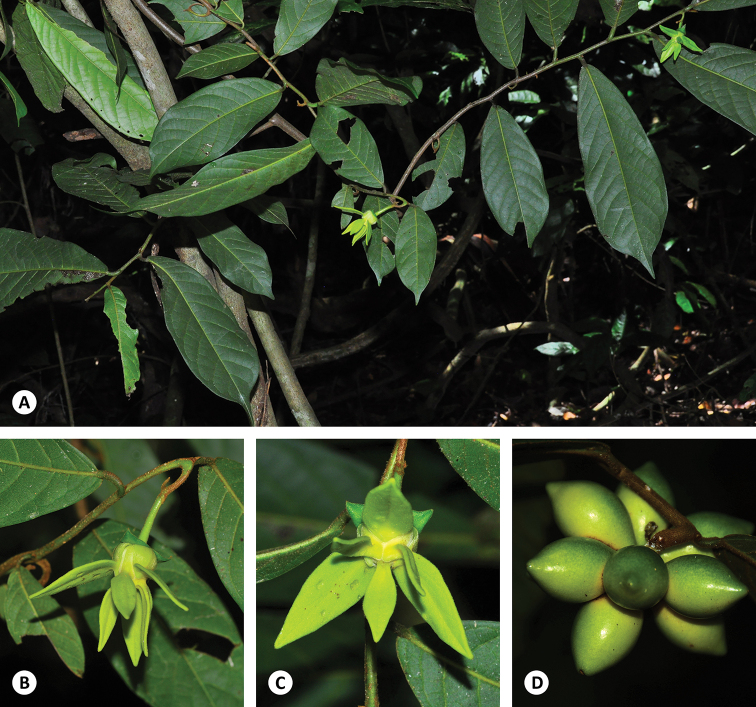
*Artabotrys insurae* sp. nov. **A** habit and leaves, showing caudate to acuminate leaf apex (*C. Leeratiwong 18-1522*) **B** oblique leaf base and inflorescence with hooked peduncle (*C. Leeratiwong 18-1522*) **C** flower, showing oblong-ovate outer petals and oblong-elliptic inner petals (*C. Leeratiwong 18-1522*) **D** fruit (*C. Leeratiwong 17-1116*). Photos: C. Leeratiwong.

#### Description.

Climbers, to ca. 5 m tall. Twigs drying light brown to greyish black, sparsely to densely erect-pubescent, becoming glabrous, epidermis non-flaky. Leaf laminas 9–19 cm long, 3.7–7.5 cm wide, oblong-elliptic to oblong-obovate, chartaceous, glabrous above except the sparsely erect-pubescent midrib and secondary veins, sparsely to densely erect-pubescent below; base oblique; apex caudate to acuminate, acumen up to 15 mm long; midrib sunken above, prominent below; secondary veins 8–13 per side, sunken to flush above, raised below; tertiary venation reticulate, visible on both surfaces; petiole 3–10 mm long, 1–1.5 mm in diameter, erect-pubescent. Inflorescences 1-flowered (rarely 2-flowered), peduncles recurved (often laterally compressed and hook-like), sparsely erect-pubescent, lateral branches condensed, pedicels 5–15 mm long, ca. 1 mm in diameter, sparsely to densely erect-pubescent. Sepals 3, free, valvate, 6–10 mm long, 5–6 mm wide, ovate, sparsely puberulent inside, densely puberulent outside, apex acute, green in vivo. Petals 6, free, valvate, sparsely to densely puberulent on both surfaces except the glabrous base inside, chartaceous, yellow in vivo, blade flat, base concave. Outer petals 3, 17–29 mm long, claw 6–8 mm wide, blade 6–12 mm wide, oblong-ovate, apex acute. Inner petals 3, 16–28 mm long, claw 4–6 mm wide, blade 3–6 mm wide, oblong-elliptic, apex acute. Stamens 20–30, ca. 2 mm long, ca. 1 mm wide, oblong, anther connective apex apiculate. Carpels 10–14, ovary ca. 3 mm long, ca. 0.5 mm wide, stigma ca. 2 mm long, cylindrical. Fruit of up to 10 monocarps borne on a subglabrous pedicel 8.5–20 mm long, 2–2.5 mm in diameter. Monocarps 23–27 mm long, 10–13 mm wide, ellipsoid, smooth, glabrous, apex weakly beaked (ca. 1 mm long), base contracted into a stipe 1.5–4 mm long, green in vivo, drying brownish black, pericarp ca. 2 mm thick. Seeds 15.5–17.8 mm long, 9.4–10.7 mm wide, 4.6–5.2 mm thick, generally smooth with wrinkled area on sides, light yellowish brown.

**Figure 4.**
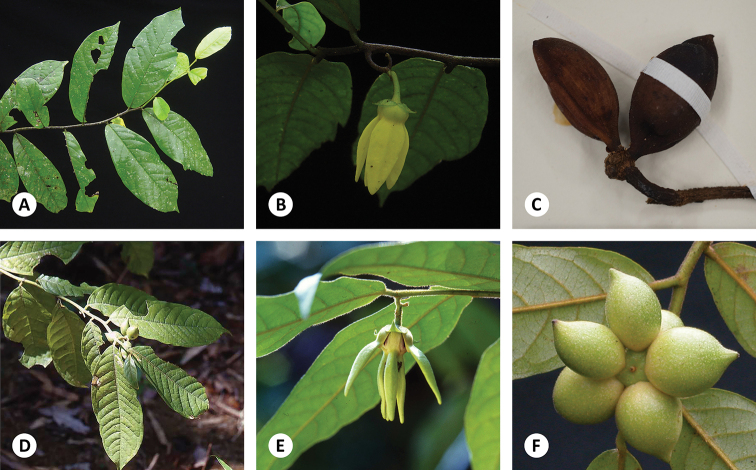
**A–C***Artabotrys insurae* sp. nov. **A** habit (*T. Insura 58*) **B** flower, showing flat petal blades (*T. Insura 58*) **C** fruit, showing weakly beaked monocarps with distinct stipes (*D.J. Middleton et al. 1487*) **D–F***Artabotrys uniflorus***D** habit **E** flower, showing three-angled petal blades **F** fruit, showing strongly beaked monocarps that are sessile. Photos: **A, B** T. Insura **C** D.M. Johnson **D–F** P. Chalermglin.

#### Phenology.

Flowering and fruiting specimens collected in August and September. Fruiting specimens also collected in February and June.

#### Distribution and habitat.

So far only known from peninsular Thailand (Fig. 6). It occurs in lowland moist and dry forests, secondary forests and forest edges at elevation 80–200 m.

#### Etymology.

The specific epithet was given in honour of Mr Tawee Insura, whose prolific collection of *Artabotrys* specimens during his MSc study led to the discovery of several new species and new records for Thailand.

#### Preliminary conservation status.

*Artabotrys insurae* is estimated to have an EOO of 15,994 km^2^ and an AOO of 20 km^2^. This species largely occurs within various Wildlife Sanctuaries, which constitute Protected Areas in Thailand. A population exists in a remnant forest adjacent to Khao Le Buddhist Temple in Songkhla; such vegetation is regarded as sacrosanct and hence would likely remain undisturbed. We suggest a status of Vulnerable [VU D2] for this species because its restricted AOO makes it susceptible to future threats such as climate change.

#### Additional specimens examined.

Peninsular Thailand. Narathiwat Province: Sukhirin District, Hala-Bala Wildlife Sanctuary, 7 September 2006, *T. Insura 75* (BK, BKF). Songkhla Province: Hat Yai District, Ton Nga Chang Wildlife Sanctuary, *Puangpen et al. N192* (QBG); idem, Ton Nga Chang Waterfall, 150 m elev., 2 February 1997, *C. Leeratiwong s.n.* (PSU); idem, 80 m elev., 12 August 2006, *T. Insura 58* (BK, BKF); Sadao District, Khao Le, 150 m elev., 16 August 2018, *C. Leeratiwong 18-1522* (PSU); Sadao District, Ton Nga Chang Wildlife Sanctuary, Pha Dam Ranger Station, 350 m elev., 2 June 2017, *C. Leeratiwong 17-1116* (PSU).

#### Notes.

This species is most similar to *A. uniflorus* from peninsular Thailand (Chumphon, Ranong, Phang-Nga and Surat Thani) in having erect-pubescent lower leaf surfaces, 1-flowered (rarely 2-flowered) inflorescences, caudate to acuminate leaf apex and relatively narrow monocarps (10–15 mm wide). Its distribution overlaps with *A. uniflorus* in Surat Thani. *Artabotrys siamensis* Miq. from Northern, Northeastern, Eastern and Southwestern Thailand is also similar in having erect-pubescent lower leaf surfaces, but is distinct due to its coriaceous leaves, cuneate leaf base, thicker petals, numerous carpels (25–29 per flower), numerous monocarps (up to 22 per fruit) and broader monocarps (15–20 mm wide).

## New records for peninsular Thailand

### Artabotrys pleurocarpus

Taxon classificationPlantaeMagnolialesAnnonaceae

Maingay ex Hook.f. & Thomson

2B1CDA47-580F-5669-8A8A-449D4C080093

Fig. 5

Artabotrys pleurocarpus Maingay ex Hook.f. & Thomson, Fl. Brit. India 1: 54 (1872). Type: Peninsular Malaysia. Malacca, 6 Feb 1868, *A.C. Maingay 3261* [Kew distribution no. 34] (lectotype K [K000381010], designated in Turner (2016) explicitly excluding material in packet; isolectotype BM [BM001014846]).

#### Distribution and habitat.

Peninsular Malaysia and peninsular Thailand (Fig. 6), in lowland rain forests.

#### Specimens examined.

Peninsular Thailand. Songkhla Province: Rattaphum District, Boripat Forest Park, 4 July 1986, *J.F. Maxwell 86-444* (A) [A00571911]; idem, 6 April 2006, *T. Insura 61* (BK, BKF). Satun Province: Thale Ban National Park, Ton Plio Falls, open area by stream, 115 m elev., 3 June 2001, *R. Pooma et al. 2004* (BKF) [SN 134816]. Trang Province: Na Yong District, Ton Pliw Waterfall, 7 April 2006, *T. Insura 62* (BK, BKF); idem, 7 April 2006, *T. Insura 63* (BK, BKF); idem, 7 April 2006, *T. Insura 64* (BK, BKF).

**Figure 5.**
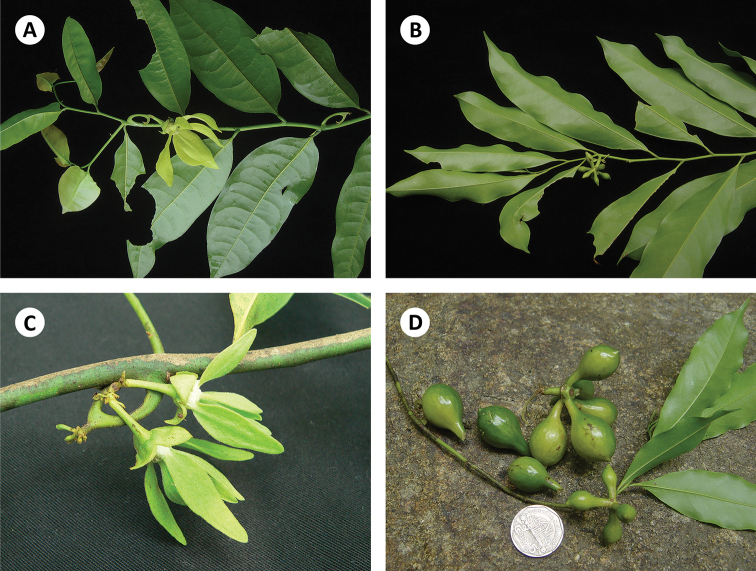
*Artabotrys pleurocarpus***A** habit and mature flower **B** habit and developing fruit **C** developing flowers on hooked inflorescence **D** fruits, showing monocarps borne on very distinct stipes. Photos: T. Insura.

#### Notes.

This species was hitherto known from Malacca, Kedah and Perak in Peninsular Malaysia (Sinclair 1955). Specimens of *A. pleurocarpus* from peninsular Thailand were formerly misidentified as *A. kurzii* Hook.f. & Thomson or identified to genus level; they were only recently re-identified during our preparation of the *Artabotrys* treatment for the Flora of Thailand. The specimens from peninsular Thailand and Peninsular Malaysia closely match one another in leaf and fruit morphology and there can be no doubt that they are conspecific. Therefore, this represents the first record of *A. pleurocarpus* in Thailand. *Artabotrys pleurocarpus* is distinct among the Thai species on account of its fruit morphology, with relatively few monocarps (up to 9 per fruit) that are prominently beaked (2–3 mm long), quite large (22–30 mm long, 15–20 mm wide) and borne on a long stipe (7–10 mm long). The fruits therefore superficially resemble those of *Polyalthia* species, but specimens can be easily assigned to *Artabotrys* if the inflorescence/infructescence hook is present. Although *A. kurzii* from Myanmar (Pegu) was previously confused with *A. pleurocarpus*, it bears little resemblance to *A. pleurocarpus*, differing in its obovate (vs. oblong-lanceolate to oblong-elliptic) leaves, mucronate (vs. caudate to acuminate) leaf apex and short petioles (1–2 mm long vs. 4–6 mm long).

### Artabotrys crassifolius

Taxon classificationPlantaeMagnolialesAnnonaceae

Hook.f. & Thomson

81D359A6-D7DB-5E86-8CB8-E8D0B890529B

Artabotrys crassifolius Hook.f. & Thomson, Fl. Brit. India 1: 54 (1872). Type: Peninsular Malaysia. Malacca, *Griffith s.n.* [EIC 426] (lectotype: K [K000607645], designated in Sinclair (1955)).

#### Distribution and habitat.

**Figure 6.**
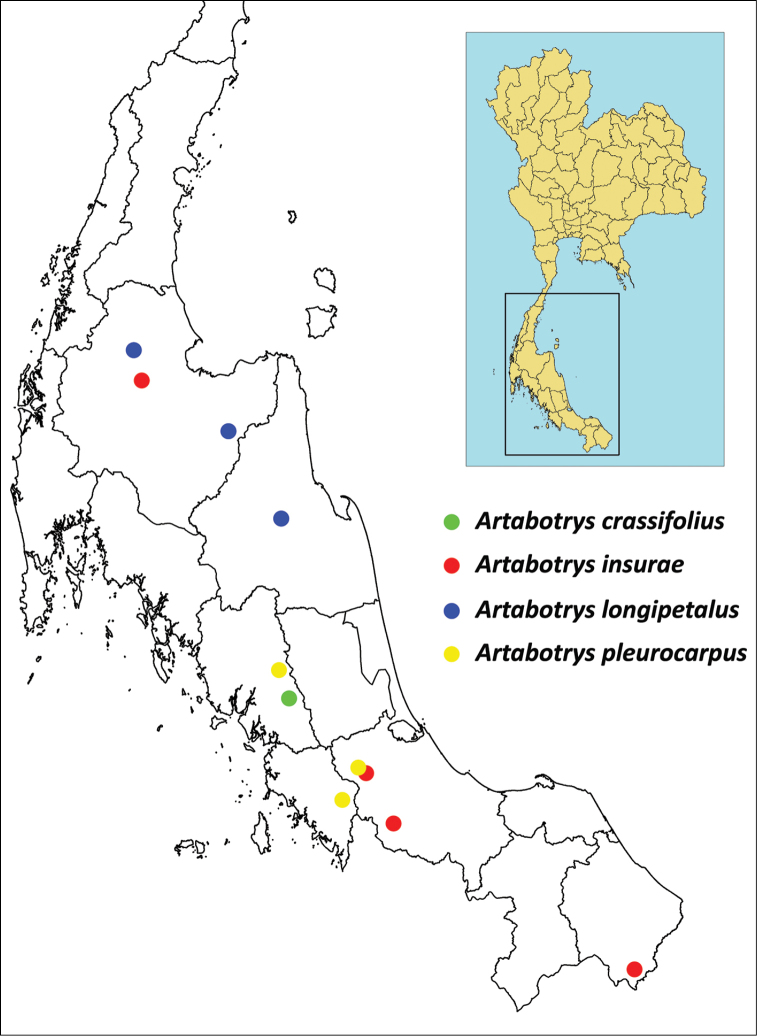
Distributions of *A. crassifolius*, *A. insurae*, *A. longipetalus* and *A. pleurocarpus* in peninsular Thailand. Only the region in peninsular Thailand is shown; adjacent areas in Peninsular Malaysia and Myanmar are removed.

Singapore, Peninsular Malaysia, peninsular Thailand (Fig. 6) and probably Myanmar (see notes), in lowland rain forests.

#### Specimen examined.

Peninsular Thailand. Trang Province: Palian District, Lam Plok Waterfall, ca. 20 m elev., 4 May 2010, *W. Eiadthong 2010-1* (BK, BKF).

#### Notes.

The protologue for *A. crassifolius* cites a specimen from Martaban in Myanmar. In addition, a regional checklist (Kress et al. 2003) and a forest flora (Kurz 1877) indicate the presence of this species in Tenasserim (Taninthayi), Myanmar. However, Turner (2015) was unable to trace the syntype or any other specimen of this species from Myanmar; our attempts to trace those specimens were likewise in vain. The occurrence of *A. crassifolius* in Myanmar therefore requires future verification. *Artabotrys crassifolius* can be distinguished from other species in Thailand as its young twigs, flower pedicels and lower surface of sepals have a dense covering of long appressed hairs that is visible with the naked eye. In Thailand, this species is currently known from a single gathering from Trang, which exhibits the unique indumentum mentioned earlier and has monocarps with shape and size matching *A. crassifolius*. The specimens of this gathering were previously filed as ‘*Artabotrys* indet’ and only recently identified for the Flora of Thailand project. Outside of Thailand, *A. crassifolius* is widespread in Peninsular Malaysia but restricted to the Central Catchment Nature Reserve and Bukit Timah Nature Reserve in Singapore.

## Key to *Artabotrys* species in Thailand

**Table:** 

1	Axillary shoots often with thorns; leaf apex retuse, truncate, rounded or mucronate (rarely acute); riparian plants	***A. spinosus***
–	Axillary shoots without thorns; leaf apex acute, acuminate or caudate (rarely or never retuse, truncate, rounded or mucronate); forest plants	**2**
2	Young twigs erect-pubescent; leaves erect-pubescent below	**3**
–	Young twigs appressed-pubescent, puberulent or glabrous; leaves glabrous or appressed-pubescent below	**5**
3	Leaves coriaceous, apex acute to acuminate (never caudate), base cuneate; petals coriaceous; carpels 25–29 per flower; monocarps up to 22 per fruit, 15–20 mm wide	***A. siamensis***
–	Leaves chartaceous, apex caudate to acuminate, base rounded or oblique (rarely cuneate); petals chartaceous; carpels 10–18 per flower; monocarps up to 12 per fruit, 10–15 mm wide	**4**
4	Leaf base rounded, rarely cuneate; petal blades three-angled; anther connective apex truncate; monocarps sessile, apex strongly beaked (2–5 mm long)	***A. uniflorus***
–	Leaf base oblique; petal blades flat; anther connective apex apiculate; monocarp base contracted into a stipe 1.5–4 mm long, apex weakly beaked (ca. 1 mm long)	***A. insurae***
5	Young twigs, flower pedicels and lower surface of sepals with a dense covering of long appressed hairs (visible with the naked eye)	***A. crassifolius***
–	Young twigs, flower pedicels and lower surface of sepals glabrous or with a sparse covering of short appressed hairs (visible with hand lens)	**6**
6	Twigs with flaky outer layer; leaf blades 21–33 cm long, tertiary venation subscalariform; inflorescence lateral branches often elongate (up to 6 cm long)	***A. byrsophyllus***
–	Twigs usually with unbroken outer layer; leaf blades 5–20 cm long, tertiary venation reticulate; inflorescence lateral branches condensed or short (up to 2.5 cm long)	**7**
7	Petals cream-white in vivo, blades terete; monocarps 1–2(–5) per fruit	***A. suaveolens***
–	Petals yellow, orange, beige, maroon or brown in vivo, blades not terete; monocarps 4–30 per fruit	**8**
8	Petals 7–14 mm long	**9**
–	Petals 15–45 mm long	**11**
9	Leaves lanceolate, base oblique or rounded; flower pedicels 3–6 mm long; outer petals ovate; monocarp base contracted into a stipe ca. 4 mm long	***A. oblanceolatus***
–	Leaves oblong-elliptic to oblong-obovate, base cuneate or decurrent on petiole; flower pedicels 7–10 mm long; outer petals deltoid; monocarps sessile or with base contracted into a stipe up to 2 mm long	**10**
10	Leaf apex acuminate to caudate; outer petal blades flat, inner petal blades spathulate; monocarp apex strongly beaked (ca. 2 mm long)	***A. spathulatus***
–	Leaf apex acute; outer petal blades undulate, inner petal blades rhomboid; monocarp apex weakly beaked (less than 1 mm long)	***A. tanaosriensis***
11	Anther connective apex apiculate	**12**
–	Anther connective apex truncate	**16**
12	Sepals ca. 3 mm long, ca. 2.5 mm wide; petal blades 1–2 mm wide	***A. tipuliferus***
–	Sepals 5–10 mm long, 5–8 mm wide; petal blades 5–18 mm wide	**13**
13	Flower pedicels 5–9 mm long	**14**
–	Flower pedicels 15–32 mm long	**15**
14	Leaf apex often caudate (sometimes acuminate); carpels ca. 10 per flower; monocarps up to 9 per fruit, 22–30 mm long, 15–20 mm wide, base contracted into a stipe 7–10 mm long	***A. pleurocarpus***
–	Leaf apex acute to acuminate (never caudate); carpels ca. 20 per flower; monocarps up to 17 per fruit, 18–20 mm long, 11–15 mm wide, base contracted into a stipe 3–4 mm long	***A. brevipes***
15	Leaves membranous; monocarp apex beaked (ca. 2 mm long), base contracted into a stipe 4–5 mm long; cultivated only	***A. hexapetalus***
–	Leaves coriaceous; monocarp apex rounded (rarely weakly beaked), base contracted into a stipe 5–12 mm long; occurs in the wild	***A. harmandii***
16	Outer petal blades 11–14 mm wide, broadly elliptic; monocarps 8–10 mm wide, apex sharply beaked ca. 5 mm long	***A. oxycarpus***
–	Outer petal blades 1–7 mm wide, ovate, lanceolate or linear; monocarps 15–28 mm wide, apex rounded or beaked up to 3 mm long	**17**
17	Outer petals ovate; monocarp base contracted into a stipe 1–3 mm long	**18**
–	Outer petals lanceolate or linear; monocarps sessile	**19**
18	Leaves chartaceous; inflorescences 10–20-flowered; carpels 15–20 per flower; monocarp apex rounded or weakly beaked; inhabits montane forests at 900–1700 m	***A. punctulatus***
–	Leaves coriaceous; inflorescences 3–5-flowered; carpels ca. 10 per flower; monocarp apex beaked ca. 3 mm long; inhabits lowland forests	***A. venustus***
19	Leaves coriaceous; petals lanceolate, apex obtuse to acute; outer petals 18–30 mm long, blade 3–5 mm wide; inner petals 18–27 mm long, blade 2–4 mm wide	***A. multiflorus***
–	Leaves chartaceous; petals linear, apex acute; outer petals 35–45 mm long, blade 1–2 mm wide; inner petals 32–40 mm long, blade 1–1.5 mm wide	***A. longipetalus***

## Supplementary Material

XML Treatment for Artabotrys longipetalus

XML Treatment for Artabotrys insurae

XML Treatment for Artabotrys pleurocarpus

XML Treatment for Artabotrys crassifolius
